# Metformin Enhances the Therapy Effects of Anti-IGF-1R mAb Figitumumab to NSCLC

**DOI:** 10.1038/srep31072

**Published:** 2016-08-04

**Authors:** Hongxin Cao, Wei Dong, Xiao Qu, Hongchang Shen, Jun Xu, Linhai Zhu, Qi Liu, Jiajun Du

**Affiliations:** 1 Institute of Oncology, Shandong Provincial Hospital Affiliated to Shandong University, Shandong University, Jinan, P.R. China; 2 Department of Chemotherapy, Cancer Center, Qilu Hospital of Shandong University, Shandong University, Jinan, P.R. China; 3 Department of Thoracic Surgery, Shandong Provincial Hospital Affiliated to Shandong University, Shandong University, Jinan, P.R. China

## Abstract

The insulin-like growth factor (IGF) signaling system plays a critical role in tumorigenesis, highlighting the potential of targeting IGF-1R as an anti-cancer therapy. Although multiple anti-IGF-1R monoclonal antibody (mAb) drugs have been developed, challenges remain in the validation of the therapeutic effects and understanding the molecular mechanism of these mAbs. Herein, we conducted a study to validate the effect of Figitumumab (CP), an anti-IGF-1R mAb, in a panel of non-small cell lung cancer (NSCLC) cell lines. We found all tested cell lines were sensitive to CP, and CP could block IGF-1R and the downstream PI3K/AKT pathway activation. Unexpectedly, we found CP could activate ERK signaling pathway in IGF-1R kinase independent manner, which we further verified was mainly mediated by β-arrestin2. We also investigated the anti-tumor effect of metformin alone as well as its combination with CP to target NSCLC. Metformin could target IGF-1R signaling pathway by attenuating PI3K/AKT and MEK/ERK signaling pathways and down-regulating IGF-1R. Finally, we found that combining metformin with CP could further induce IGF-1R down-regulation and was more effective to target NSCLC cells. Our data suggests the combining of metformin with CP has additive therapeutic value against NSCLC.

The insulin-like growth factor (IGF) signaling system plays critical roles in tumor cell proliferation, apoptosis, malignant transformation, angiogenesis and cell motility^1^,^2^,^3^,^4^. In lung cancer, over-expression of IGF-1 and/or IGF-1R was associated with poor prognosis and survival^5^,^6^,^7^,^8^. IGF-1R targeting therapy has become a highly attractive area in anti-cancer drug development during the last decade^9^. Antibodies against IGF-1R were designed to specifically block ligand-induced receptor activation by completing with ligands, and thus induce receptor internalization/degradation and cell signaling abrogation^10^. Several preclinical and clinical studies have demonstrated the efficacy of these anti-IGF-1R mAbs in cancer therapy^11^,^12^; however, challenging remains because some anti-IGF-1R mAbs failed to shown similar therapeutic effects in clinical trials, with hyperglycemia being one of the most frequent side effects^13^,^14^,^15^.

CP (Figitumumab, CP-751,871), a monoclonal anti-IGF-1R antibody, has been shown to suppressed tumor initiation and progression in some preclinical studies^16^,^17^,^18^. Phase I, II trials on CP showed some promising results, with well-tolerance and mild adverse events^19^,^20^. As to NSCLC, Phase II trial showed CP was safe and effective, but a phase III trial in advanced NSCLC with CP showed significantly more side effects and less efficacy, which unfortunately resulted in the discontinuation of the trial^21^. In order to decide whether we should resume the clinical trial on CP, it is important for us to better understand the molecular mechanism of CP, which might help us to stratify the NSCLC patients and minimize its side effects. In addition, it is critical to study the combination of CP with other drugs that could potentially enhance its therapeutic effects against NSCLC, and thus could encourage the enrollment of patients into the trial.

Metformin (1, 1-dimethylbiguanide) is drawing increasing attention for its potential anti-neoplastic effects. Several clinic studies have observed intriguing results that metformin is associated with risk and/or mortality in many cancer types, including lung cancer^22^,^23^. Meanwhile, increasing experimental data have revealed metformin’s anti-cancer properties including inhibiting cancer cell proliferation, migration, invasion and metastasis^24^,^25^,^26^. Since many cancer cells are characterized with a constitutive high glucose uptake rate^27^, the “calorie restriction mimetic” activities of metformin are deemed to among the factors that contribute to its inhibitory effects on cancer growth and development^28^,^29^,^30^. Therefore, metformin is incorporated into current studies for cancer cell metabolism therapeutic approaches. At the molecular level, the activation of LKB1/AMPK/mTOR pathway and the inhibition of insulin-induced bio-cellular activities are investigated to exert its anti-neoplastic effects^31^.

Beyond the above, the emerging effects of metformin on IGF system capture our attention. Werner *et al*. has demonstrated that metformin could down-regulate the IGF-1R signaling pathway in a panel of uterine serous carcinoma cell lines, leading to the inhibition of cell proliferation and migration regardless of the p53 status^32^. In another study, metformin is reported to diminish the phosphorylation of a series of receptor tyrosine kinase (RTKs) and the down-stream PI3/AKT/mTOR and Ras/Raf/MEK/ERK pathways in lung tissues in an AMPK independent pattern in liver IGF-1–deficient (LID) mice model with NNK-induced lung carcinogenesis^33^.

β-arrestins are documented to function as E3 ubiquitin ligase adaptors thus mediate ubiquitylation of seven-transmembrane receptors (7TMRs), which result in receptor endocytosis, recycling or degradation^34^,^35^. Recent studies have broadened the functional scope of β-arrestins in receptor internalization to the field of RTKs, such as TGF-β and IGF-1R^36^,^37^. The concept of “biased agonist” has cut a figure in the field of 7TMRs, and biased ligands and receptors have been identified that preferentially signal through either G protein- or β-arrestin-biased pathways^34^. Biased agonists give intriguing possibility of directing cellular signaling with unprecedented precision and specificity and facilitate the development of new types of drugs with fewer side effects^35^,^38^.

Our group aims at testing the sensitivity of NSCLC cells to CP and further testing the effect of CP on signaling pathways and receptor down-regulation. In particular, we discussed the role of β-arrestins in regulating cell signaling and therapeutic effect. Furthermore, we tested the therapeutic enhancement effect of metformin when combining with CP to treat NSCLC. We summarized the theoretical basis that support our hypothesis as following: (1) the caloric restriction effect and other anti-neoplastic mechanisms make metformin good choice in cancer therapy (2) anti-IGF-1R mAbs targeting therapy is promising approach against cancer, including NSCLC (3) inhibition effects on IGF-1R signaling system highlighting metformin as a candidate as an adjuvant in anti-IGF-1R system therapy (4) anti-IGF-1R mAb targeting therapy may benefit from metformin for its hypoglycemic property by reducing side effects. In summary our results help to better understand the mechanism and side effects of CP when using it to target NSCLC and we demonstrated the potential of combining CP with metformin as a novel NSCLC therapeutic strategy.

## Results

### Sensitivity to CP treatment in NSCLC cell lines

**Figure 1:**
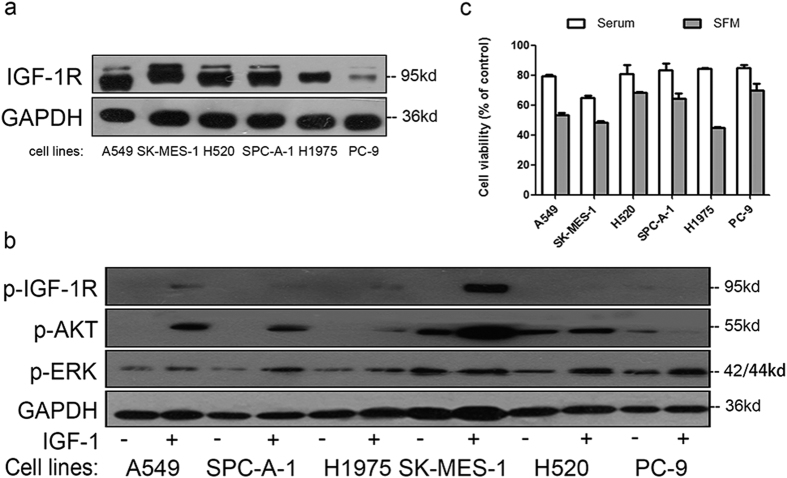
IGF-1R expression and sensitivity to CP treatment in NSCLC cell lines. (**a**) NSCLC cell lysates were prepared and IGF-1R were detected by WB. (**b**) Cells were starved for 12h and stimulated with 50 ng/ml IGF-1 for 10 min. p-IGF-1R, p-ERK, p-AKT and GAPDH were detected by WB. (**c**) Cells were treated with 100 ng/ml of CP for 48 h with presence or absence of serum. Cell viability was tested through MTT. The number of viable cells following CP treatment was presented as percentage of untreated cells. Data were presented as mean ± S.E.M.

Firstly, we investigated the IGF-1R expression level in a panel of NSCLC cell lines, including A549, SK-MES-1, H520, SPC-A-1, H1975 and PC-9 (Fig. 1a). IGF-1R was expressed in all tested NSCLC cell lines, although the exact expression level was variable, with A549 and PC-9 displaying the highest and lowest levels, respectively. We then stimulated these cells with IGF-1, a complete IGF-1R agonist. As shown in Fig. 1b, IGF-1 could induce IGF-1R phosphorylation and activation of downstream RAS/RAF/MEK/ERK and PI3K/AKT signaling pathways in most cell lines, and the level of activation was generally correlated with the amount of IGF-1R in the cell. These results suggested that IGF-1R signaling pathway was responsive in all the cell lines we used.

Subsequently, we investigated the sensitivity of these NSCLC cells to anti-IGF-1R mAb CP (100 ng/mL CP, whose molar concentration is 10-fold less than 50 ng/mL IGF-1). As demonstrated in Fig. 1c, cell viability decreased in all NSCLC cell lines after CP treatment, ranging from 64.7% in SK-MES-1 to 85.0% in PC-9 under serum added medium conditions, and from 45.0% in H1975 and 70.1% in PC-9 under serum free medium (SFM) conditions. CP was originally designed to compete with ligands for binding of IGF-1R, thus preventing the ligand-induced signaling pathway activation. This idea was supported by the inhibitory effect of CP seen in NSCLC cell lines in serum-added medium (Fig. 1c). However, it was surprising to see that CP had more inhibitory effects in cells under SFM conditions. It is possible that besides being an IGF-1 competitor, CP might have another IGF-1R dependent mechanism to inhibit cell proliferation and/or promote cell death.

### β-Arrestins regulate IGF-1 but not CP-induced IGF-1R down-regulation

**Figure 2:**
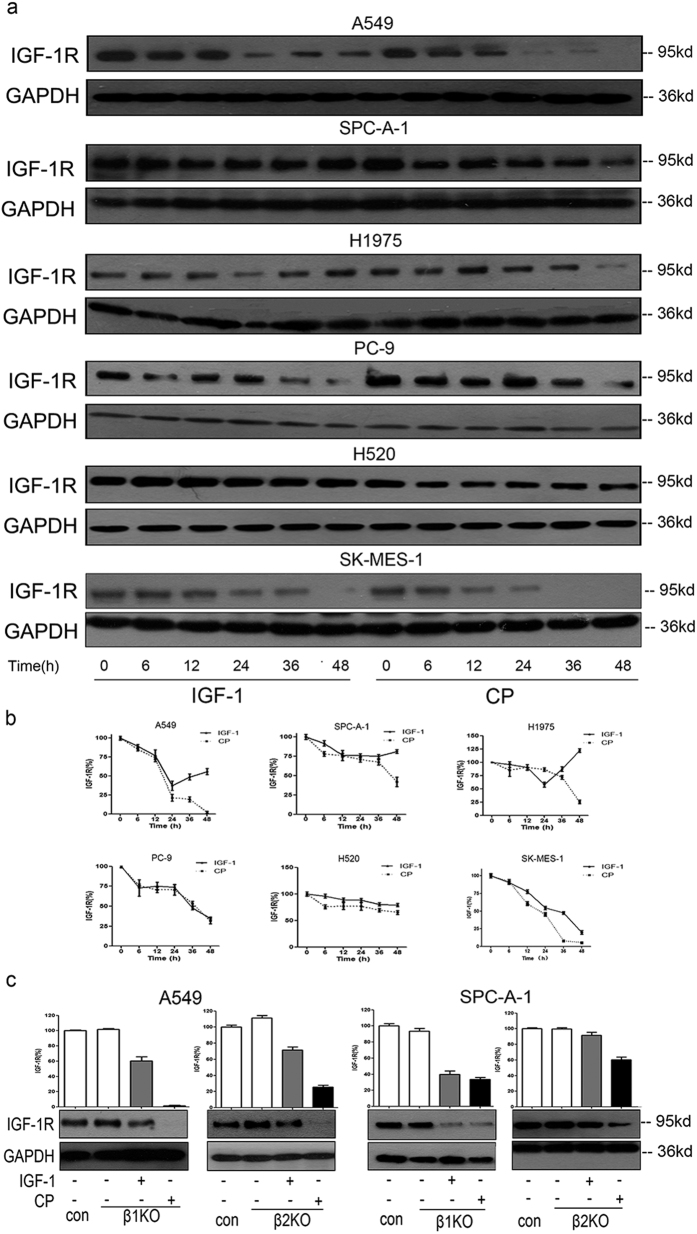
CP could induce IGF-1R down-regulation. (**a**) Cells were starved for 12 hs, then treated with 50 ng/ml IGF-1 or 100 ng/ml CP for a serious of time points (0, 6, 12, 24, 36, 48 h). Cell lysates were prepared and the level of IGF-1R was detected WB. (**b**) Intensity of the bands for IGF-1R at different time points in Fig. 2a were quantified by densitometry, normalized to GAPDH, and displayed as a percentage of the intensity at 0 h. Data were presented as mean ± S.E.M. (**c**) A549 and SPC-A-1 cells were knocked down for either β-arrestin 1 or β-arrestin 2. Cells were then starved for 12 h, treated with 100 ng/ml CP or 50 ng/ml IGF-1 for 24 h. Cells transfected with scrambled siRNA as control. IGF-1R and GAPDH were detected via WB. The intensity of the bands was presented as mean ± S.E.M.

Multiple RTK-targeting mAbs were documented to have a delayed effect on the down-regulation of corresponding receptors. We processed a series of experiments to verify the receptor down-regulation property of CP and investigated its possible mechanisms. In order to get the pure effects of CP without the competition of IGF-1 or other growth factors that are normally presented in serum, all experiments were performed in SFM condition. As shown in Fig. 2a,b, both IGF-1 and CP could induce IGF-1R down-regulation in a time-dependent manner. The IGF-1R down-regulation effect was stronger and more sustained after CP treatment in most cell lines, especially at later time points, while in multiple IGF-1 treated cells (A549, H1975 and SPC-A-1) the expression of IGF-1R rebounded at around 48h. Since the molar concentration of CP was 10-fold less than that of IGF-1, our data suggested that CP is more effective in inducing IGF-1R down-regulation.

We tried to investigate if β-arrestins regulate CP-induced IGF-1R down-regulation. We used siRNA to knock-down (KD) specific β-arrestin isoform (β-arrestin1 and β-arrestin2) in A549 and SPC-A-1 cell lines. As shown in Fig. 2c, both β-arrestin1 and β-arrestin2 are involved in IGF-1-induced receptor down-regulation in A549 cells, while β-arrestin2 is the main isoform that regulates IGF-1-induced receptor down-regulation in SPC-A-1 cells; however, neither β-arrestin1 KD nor β-arrestin2 KD could rescue CP-induced IGR-1R down-regulation. These data implies that CP-induced IGF-1R endocytosis and down-regulation is β-arrestins independent and thus is different from that of IGF-1.

### CP could induce β-arrestin–dependent ERK activation and inhibiting ERK activation could increase the therapeutic effect of CP against NSCLC

**Figure 3:**
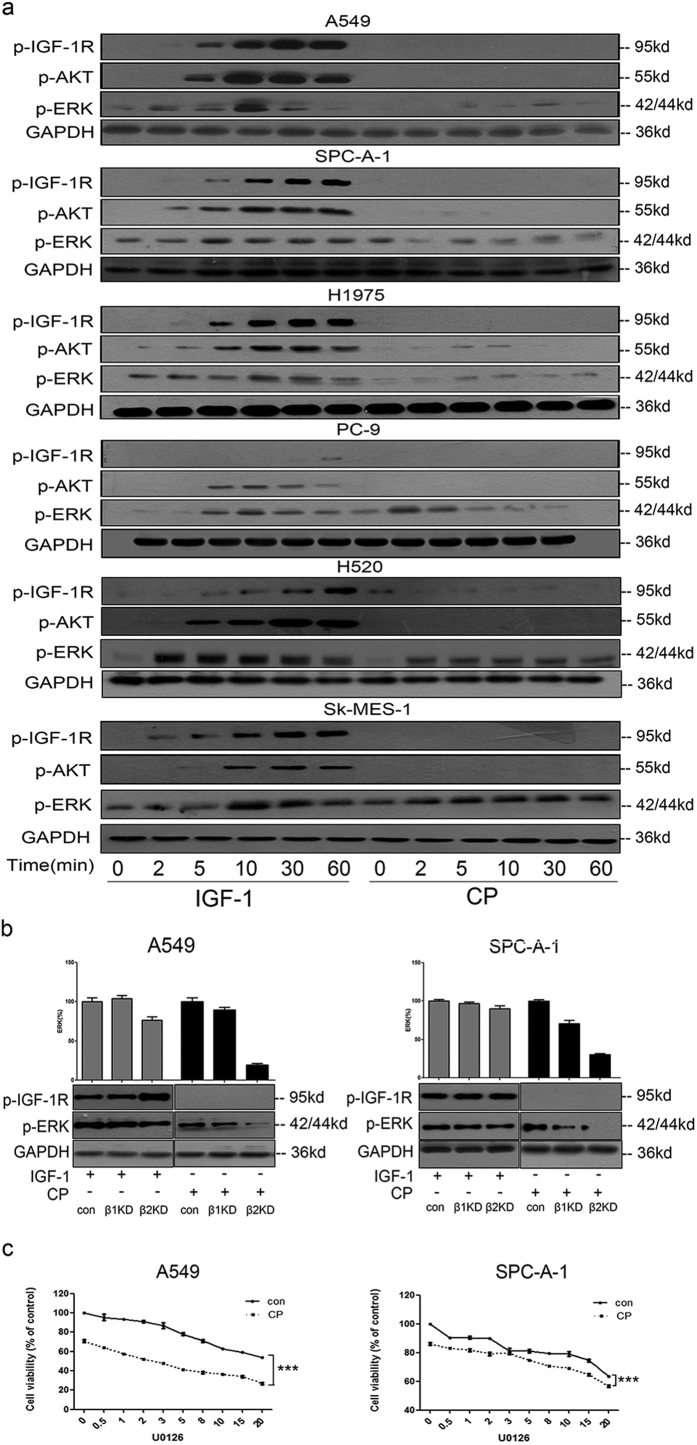
CP could induce β-arrestin–dependent ERK activation and inhibiting ERK activation could increase the therapeutic effect of CP. (**a**) Cells were starved for 12 h and treated with 50 ng/ml IGF-1 or 100 ng/ml CP for a series of time points (0, 2, 5, 10, 30, 60 min). Cell lysates were analyzed via WB for p-IGF-1R, p-AKT, p-ERK and GAPDH. (**b**) A549 and SPC-A-1 cells were knocked down for either β-arrestin1 or β-arrestin 2. Cells were then starved for 12 h, treated with 100 ng/ml CP or 50 ng/ml IGF-1 for 10 min. Cells transfected with scrambled siRNA as controls. p-IGF-1R, p-ERK and GAPDH were detected via WB. The intensity of the bands was presented as mean ± S.E.M. (**c**) A549 and SPC-A-1 cells were treated with U0126 (0, 0.5, 1, 2, 3, 5, 8, 10, 15, 20 μM) for 60 min, and then incubated with or without 100 ng/ml CP for 48 h. Cell viability was tested via MTT. The number of viable cells following treatment is presented as percentage of the untreated cells. Data were presented as mean ± S.E.M.

We further investigated the effects of CP on IGF-1R signaling pathways in NSCLC cells and compared it with IGF-1 stimulation (Fig. 3a). As a full agonist, IGF-1 could induce IGF-1R phosphorylation and downstream signaling pathways activation within 5 min. The activation effects of IGF-1 showed in a time depended pattern, peaking around 10 to 30 min and lasting for at least 60 min. In contrast, p-IGF-1R and p-AKT were undetectable after CP treatment. To our surprise, CP could induce ERK phosphorylation in most NSCLC cell lines, although the ERK activation level was still lower than that induced by IGF-1. Since IGF-1R was kept un-phosphorylated (under the sensitivity of Western Blot) under CP treatment, it suggested that there might be another IGF-1R kinase independent mechanism to mediate the phosphorylation of ERK.

There have been several studies demonstrating the novel role of β-arrestins to regulate the G protein–coupled receptors (GPCRs) signaling pathway^39^. We investigated the hypothesis that β-arrestins play a role in IGF-1R-independent ERK activation. As shown in Fig. 3b, knockdown of either β-arrestin 1 or β-arrestin 2 did not interfere the IGF-1 induced ERK phosphorylation, suggesting different mechanisms of downstream pathways activation between IGF-1R and GPCRs. Intriguingly, knockdown of β-arrestin 1 moderately decreased CP-induced ERK phosphorylation while knockdown of β-arrestin 2 almost completely inhibited the CP-stimulated activation of ERK (Fig. 3b). Our results suggested that CP could induce IGF-1R phosphorylation independent activation of ERK, and this process is mediated through β-arrestins, mainly β-arrestin 2.

Due to the surprising ERK activation from CP treatment, we suspected that it might compromise the therapeutic effect of CP to target NSCLC. We then tried to use U0126, a MEK inhibitor, to block ERK activation. U0126 was added 60 min prior to CP treatment in A549 and SPC-A-1 cell lines. As shown in Fig. 3c, U0126 treatment could further inhibit the proliferation of CP-treated NSCLC cells, with higher U0126 having more significant inhibition effect. These results suggested that accidental ERK activation might be one of the main side effects of CP, and combing CP with ERK inhibitors could potentially enhance the efficacy of CP therapy to target NSCLC.

### Anti-proliferation effect of metformin alone and in combination with CP in NSCLC cell lines

**Figure 4:**
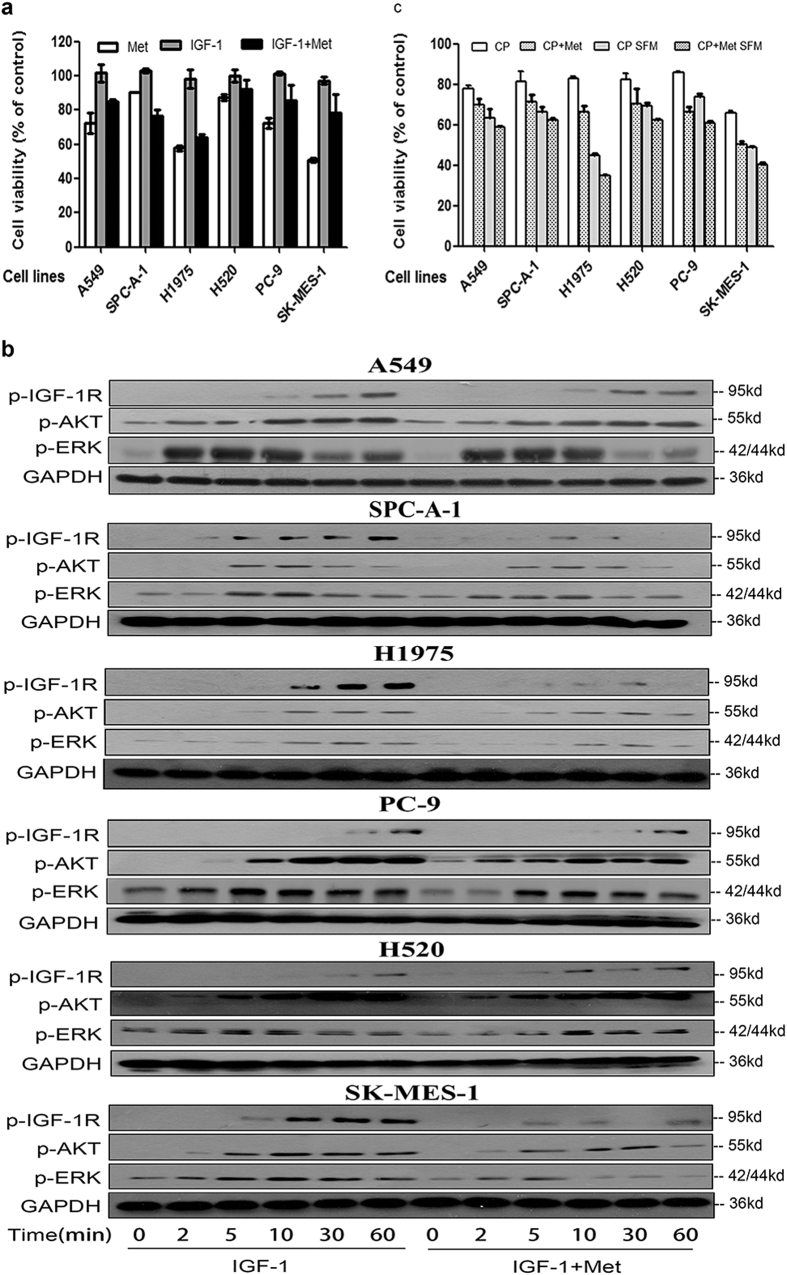
Metformin inhibits NSCLC through IGF-1R signaling pathway. (**a**) Cells were treated 48h with metformin (3 mM) alone, IGF-1 (50 ng/ml) alone, or IGF-1 plus metformin. Cell viability was tested via MTT. The number of viable cells following treatments is presented as percentage of untreated cells. Data were presented as mean ± S.E.M. (**b**) After 12 h starvation, A549 and SPC-A-1 cells were treated with or without 3 mM metformin for 1 h. Cells were then treated with 50 ng/ml IGF-1 for a series of time points. Cell lysates were analyzed via WB for p-IGF-1R, p-AKT, p-ERK and GAPDH. (**c**) Cells were pre-treated with 3 mM Metformin for 1 h, and then 100 ng/ml CP was added with presence or absence of serum. MTT was conducted to test cell viability after 48 h of incubation. The number of viable cells following treatment is presented as percentage of untreated cells. Data were presented as mean ± S.E.M.

Having shown that CP could be potentially used as a drug to target NSCLC and its side effect of accidental ERK activation, we then tried to investigate if CP could coordinate with metformin to treat NSCLC. Firstly, we investigated the inhibition effects of metformin alone on a panel of NSCLC cell lines. As shown in Fig. 4a, cell viability decreased in all NSCLC cell lines that were tested, ranging from 50.6% in SK-MES-1 to 90.5% in SPC-A-1 cell lines. In addition, in most cell lines IGF-1 could partially rescue the growth inhibitory effects of metformin, implying that simultaneously inhibiting IGF-1R might further improve the anti-tumor effect of metformin. We further tested the effects of metformin on the IGF-1R signaling pathway. As demonstrated in Fig. 4b, metformin could significantly attenuate the IGF-1 induced activation of IGF-1R, AKT and ERK in a panel of NSCLC cell lines, which could at least partially explain the antitumor effects of metformin in Fig. 4a.

Having demonstrated that both metformin and CP target IGF-1R signaling pathway, the next question is whether combining CP and metformin could have additive effects on NSCLC cell lines. Consistent with previous results, CP could effectively inhibit cell proliferation in NSCLC cell lines and CP is more effective in SFM condition than serum-added conditions (Fig. 4c). In addition, the combination of metformin with CP could further decrease the cell survival, with cell viability ranging from 50.6% in SK-MES-1 to 71.5% in SPC-A-1 under serum added conditions while ranging from 34.8% in H1975 to 62.3% in H520 under the SFM conditions. Our result suggested that CP and metformin have additive therapeutic effects to target NSCLC cell lines. Even though both CP and metformin target IGF-1R signaling pathway, the combination might further inhibit IGF-1R activation, and/or either metformin or CP have additional anti-tumor effects that are not overlapped with each other.

### Metformin could promote the CP-induced IGF-1R down-regulation

**Figure 5:**
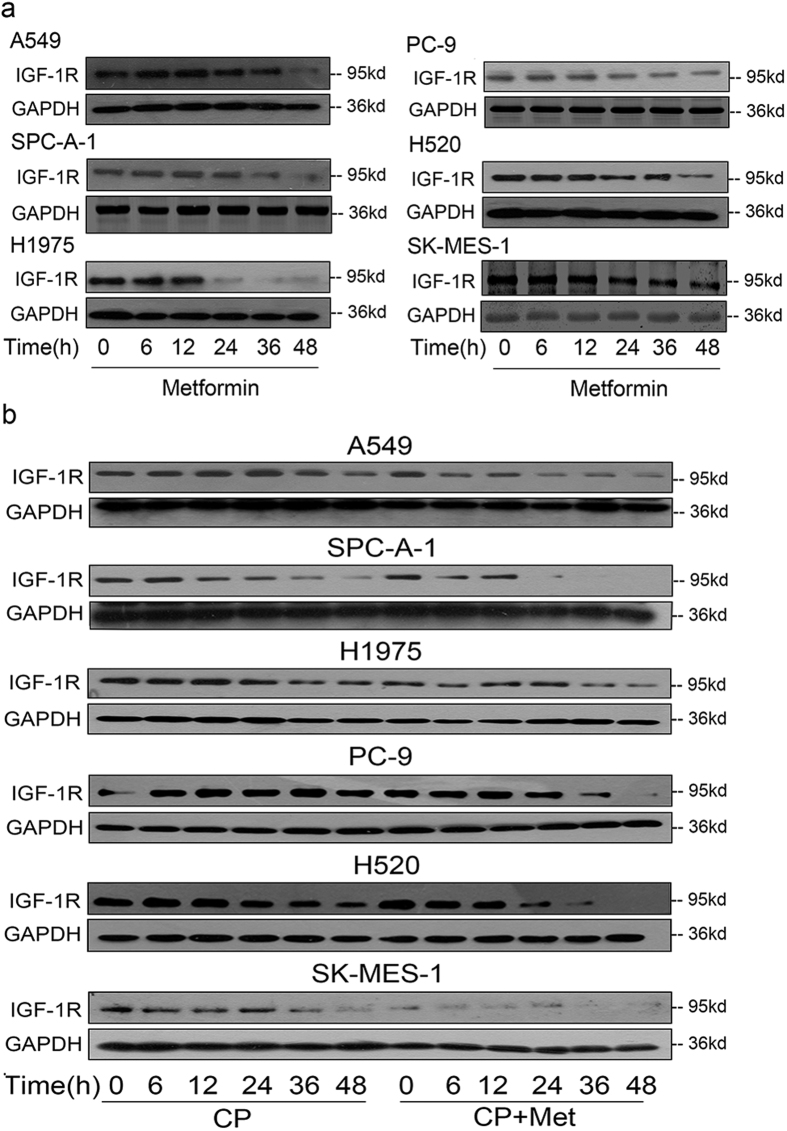
Metformin could promote the CP-induced IGF-1R down-regulation. (**a**) 3 mM Metformin was added to cells for a series of time points (0, 6, 12, 24, 36, 48 h). The expression level of IGF-1R and GAPDH were detected through WB. (**b**) Cells were starved for 12h, 3mM Metformin was added to cells for 1h, then 100 ng/ml CP was added for a series of time points (0, 6, 12, 24, 36, 48 h). The expression level of IGF-1R and GAPDH were detected through WB.

To further elucidate the additive therapeutic effects of CP and metformin, we tried to check the IGF-1R expression level after drug treatment in NSCLC cell lines. As shown in Fig. 5a, a time-dependent down-regulation of IGF-1R was seen in all the tested cell lines, which was also seen under CP treatment (Fig. 2a). We then assessed the receptor down-regulation after combination metformin and CP treatment. As demonstrated in Fig. 5b, CP plus metformin could further down-regulate IGF-1R compared with CP only group. Therefore, the additive IGF-1R down-regulation could at least partially explain the additive therapeutic effect of combining CP and metformin to target NSCLC.

### IGF -1R and β-arrestin 2 are highly expressed in NSCLC tumor tissues

**Figure 6:**
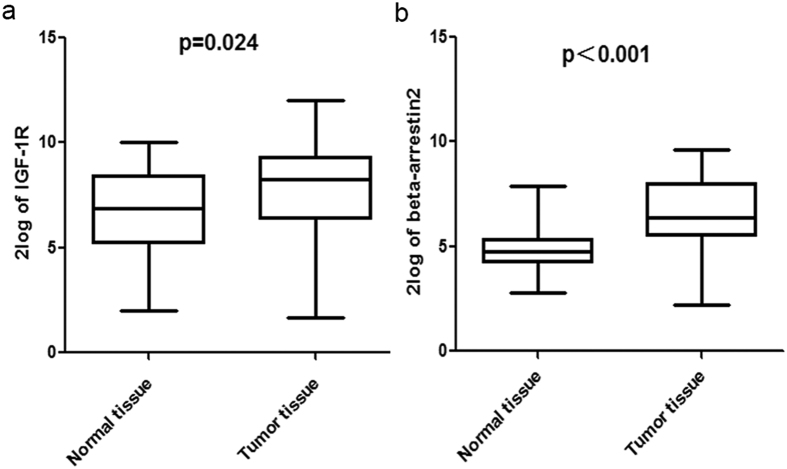
IGF -1R and β-arrestin 2 mRNA expression in NSCLC tumor tissues. (**a**) IGF -1R mRNA expression levels in tumor tissues and normal tissues. Data was shown as 2log of the values. (**b**) β-arrestin 2 mRNA expression levels in tumor tissues and normal tissues. Data was shown as 2log of the values.

Based on the above results, we recognized the importance of IGF -1R and the corresponding signaling pathway in NSCLC. And the role of β-arrestins in receptor down-regulation and signaling transduction implied the potential of β-arrestins as a new target. We tested mRNA levels of IGF -1R and β-arrestin2 in tumor tissues and the corresponding normal tissues of 35 patients from Shandong Provincial Hospital Affiliated to Shandong University by qRT-PCR. The clinical characters of the patients are described in Table 1. As shown in Fig. 6a, the expression of IGF -1R mRNA was higher in NSCLC tumors than the normal tissue (p = 0.024), confirming the critical role of IGF -1R in carcinogenesis. And β-arrestin2 mRNA level was higher in tumor tissues than normal tissues (p < 0.001) (Fig. 6b), suggesting the active of β-arrestin2 in NSCLC.

## Discussion

In the current study, we tested the effects of CP on a panel of NSCLC cell lines and all tested cell lines were sensitive to CP treatment in both serum-added and serum-free medium. On one hand, the inhibition of cell proliferation in serum-added medium verified that CP could block IGF-1R signaling by competing with IGF-1. On the other hand, the proliferation inhibition effects observed in serum-free medium implied that there might have IGF-1R phosphorylation independent mechanisms that account for the therapeutic effect of CP.

We verified IGF-1R down-regulation effect of CP in NSCLC cells and our data showed the roles of β-arrestins in IGF-1 and CP induced IGF-1R down-regulation are different. β-arrestins serves as E3 ligase adaptors to mediate ubiquitylation of 7TMRs and thus scaffold receptor endocytosis and traffick intracellular itinerary of the receptors, and this pattern was also observed in IGF-1R that β-arrestin1 participated in IGF-1R ubiquitylation and down-regulation^39^,^40^. The exact role of β-arrestin1 and β-arrestin2 seems to be different depending on different cell types and receptors^34^. Also, there’s study illustrating that there are different ubiquitylation sites on β-arrestin2 which would govern its interaction with different receptors and subsequent internalization and downstream signaling^41^.

We investigated the effects of CP on IGF-1R signaling pathway and unexpectedly found CP could provoke ERK activation without IGF-1R phosphorylation, which might complain some of the side effects observed in CP clinical trials. Furthermore, we found that CP-induced ERK activation was mediated through β-arrestin2. Based on above, we would like to consider CP as a β-arrestin-biased agonist to IGF-1R. β-arrestins have been shown to scaffold with c-Src and lead to subsequent ERK1/2 activation of in β2AR^42^, and massive *in vitro* and *in vivo* data support β-arrestins as a signaling transducer^34^,^43^,^44^,^45^. Moreover, the β-arrestins-dependent process was reported to be temporally slower onset, which is consistent with our data that CP-induced p-ERK was both weaker and slower than that of IGF-1. Signaling of the two biased arms is pharmacologically distinct, that is, one biased signaling pathway may translate into favorable physiological effects whereas the other appears to result in unbeneficial results^46^,^47^. This notion highlights the potential to improve therapeutic outcomes by preferring or avoiding specific signaling arms. When it comes to CP-induced signal activation in NSCLC, the β-arrestin2-biased ERK signaling could be inhibited by U0126, suggesting the potential of controlling the biased signaling to enhance therapeutic effect of CP in the future. A related experiment was done by Leong *et al*., who demonstrated that the inhibition of MEK signaling with U0126 might help to overcome IGF-1R/IR resistance *in vitro* and *in vivo* in colorectal cancer^48^. Although several studies have demonstrated that β-arrestins served as scaffolding proteins to regulate specific components of the MAPK cascade, their exact molecular functions vary depending on specific agonists, receptors and cell types^49^,^50^. The details of the CP-induced ERK signaling need to be further investigated.

We then assessed the anti-proliferation effects of metformin on a panel of NSCLC cell lines, and our results suggested that the anti-tumor effect of metformin was at least partially though the inhibition of IGF-1R signaling pathway. We also found that metformin could down-regulate IGF-1R and attenuate IGF-1R phosphorylation and the downstream PI3K/AKT and Ras/Raf/MEK/ERK pathway activation after IGF-1 stimulation, which further echoed our hypothesis. Our finding is agreed with previous studies that treatment with metformin inhibit cell viability via inhibition of AKT signaling in glioblastoma^51^. Meanwhile, there are studies demonstrating the effect of metformin on ERK signaling pathway^33^,^52^. Because recent studies on metformin mainly focus on AMPK signaling pathway^25^,^31^, our findings help to strengthen the role of metformin on IGF-1R signaling pathway, making metformin a promising candidate in anti-cancer therapy. Actually, there have been studies reporting the use of metformin to improve the clinic prognosis in patients who underwent regular chemotherapy with esophageal adenocarcinoma, rectal cancer and other cancers^53^,^54^. Some other studies also investigated the synergistic anti-cancer effects of metformin when combining with BRAF or other tyrosine kinase inhibitors in cell lines and animal models^15^,^55^. Herein, we tested the combination of metformin with CP treatment and we observed an additive anti-cancer effects when targeting NSCLC cell lines, which we think is partially mediated through IGF-1R down-regulation and signaling attenuation.

## Conclusion

Although CP can block IGF-1R downstream PI3K/AKT signaling pathway through ligand competition, CP could also provoke an IGF-1R phosphorylation independent ERK activation mediated via β-arrestin2, which may probably influence its therapeutic effect. CP can down-regulate IGF-1R, which would contribute to the therapeutic benefits. The adding of U0126 and metformin may enhance the therapeutic effect of CP to target NSCLC.

## Materials and Methods

### Reagents

IGF-1Rβ, p-IGF-1β, p-Erk1/2, p-Akt were purchased from Cell Signaling Technology. β-arrestin1 and β-arrestin2 were from Abcam. GAPDH was from Santa Cruz Biotechnology. U0126 was from Calbiochem. IGF-1 was from Sigma. Figitumumab was provided by Pfizer.

### Cell Culture

A549, PC-9, H520, SPC-A-1, NCI-H1975 and SK-MES-1 cell lines were purchased from ATCC, SK-MES-1 was cultured in EMEM medium, the other cell lines were cultured in RIPM H1640 medium supplemented with 10% fetal bovine serum (FBS), and grown in an atmosphere of 5% CO_2_ /95% humidified air at 37 °C. Cell culture media was from Thermo Scientific Hyclone and FBS was obtained from Life Technologies Gibco.

### Transfection

The siRNA of β-arrestin-1(ccaguccaaauggaaagcutt)(257) and β-arrestin-2(cgagccuucugugcuaaautt) (731) were obtained from GenePharma (Shanghai, China). For the silencing of β-arrestin-1 or β-arrestin-2, transient transfection of siRNAs (30nM) targeting human β-arrestin-1 or β-arrestin-2, negative control (scrambled sequence), was performed using LipofectamineRNAiMAX (Invitrogen) according to the manufacturer’s protocol. Each knockdown experiment described herein was detected for specific reduced expression of β-arrestins (75–90%) 48 h later.

### Western Blot Analysis

Protein samples were dissolved in lithium dodecyl sulfate (LDS) sample buffer (Invitrogen) and equal amounts of samples were separated by SDS/PAGE. We have described the detail protocol previously^56^.

### Densitometry Analysis

Band intensity was measured by ImageJ 1.63 (US National Institutes of Health, Bethesda, Maryland, USA,) and displayed relative to band intensity of the stated loading control.

### Cell Viability Assay/Cell proliferation assay

Cell proliferation was measured with MTT. Cells were incubated in 96-well tissue culture plates, and after appropriate treatment time, MTT was added to a final concentration of 1 mg/mL, and the reaction mixture was incubated for 4 hours at 37 °C. The resulting crystals were dissolved in DMSO and the absorbance was read at 560 nm. Each experiment was repeated thrice, with cells without any stimulation as controls.

### Patient tissues and qRT-PCR

The frozen tissues were from patients with primary NSCLC who had undergone a surgical resection in Shandong Provincial Hospital. This study was reviewed and approved by the Ethical Committee of Shandong Provincial Hospital, and written informed consent was given by participants. All experiments were performed in accordance with relevant guidelines and regulations. Total RNA was obtained from NSCLC frozen tumor tissues and the corresponding pare-neoplastic normal tissues, pericarcinous tissues of lung cancer and cell lines using TRIzol reagent (Invitrogen), according to the manufacturer’s instructions. RNA concentration was determined spectrophoto metrically, and integrity was checked by gel electrophoresis. mRNA was converted into cDNA using PrimeScript RT reagent Kit with gDNA Eraser (Takara, Japan). Afterwards, each cDNA was amplified with SYBR Premix Ex Taq (TliRNaseH Plus, Takara, Japan) and analyzed by Roche LightCycler 480II.

### Statistical Analysis

Statistical analysis was done using the Student t test or two-way ANOVA to correct for multiple comparisons, as appropriated. All statistical tests were two-sided and were done using SPSS software 19.0 (SPSS Inc., Chicago, IL).

## Additional Information

**How to cite this article**: Cao, H. *et al*. Metformin Enhances the Therapy Effects of Anti-IGF-1R mAb Figitumumab to NSCLC. *Sci. Rep.* **6**, 31072; doi: 10.1038/srep31072 (2016).

## Figures and Tables

**Table 1:** The characters of the patients.

Parameters	Patients (number)
Gender	
Male	27
Female	8
Age	
<60	10
≥60	25
Smoking history	
No	8
Yes	27
Histotype	
Adenocarcinoma	14
Squamous carcinoma	21
Pathological tumor stage	
T1/2	31
T3	4
Lymph node metastasis	
Negative	19
Positive	16
